# A Global Trend towards the Loss of Evolutionarily Unique Species in Mangrove Ecosystems

**DOI:** 10.1371/journal.pone.0066686

**Published:** 2013-06-21

**Authors:** Barnabas H. Daru, Kowiyou Yessoufou, Ledile T. Mankga, T. Jonathan Davies

**Affiliations:** 1 African Centre for DNA Barcoding, University of Johannesburg, Johannesburg, South Africa; 2 Department of Biology, McGill University, Montreal, Canada; University of Florida, United States of America

## Abstract

The mangrove biome stands out as a distinct forest type at the interface between terrestrial, estuarine, and near-shore marine ecosystems. However, mangrove species are increasingly threatened and experiencing range contraction across the globe that requires urgent conservation action. Here, we assess the spatial distribution of mangrove species richness and evolutionary diversity, and evaluate potential predictors of global declines and risk of extinction. We found that human pressure, measured as the number of different uses associated with mangroves, correlated strongly, but negatively, with extinction probability, whereas species ages were the best predictor of global decline, explaining 15% of variation in extinction risk. Although the majority of mangrove species are categorised by the IUCN as Least Concern, our finding that the more threatened species also tend to be those that are more evolutionarily unique is of concern because their extinction would result in a greater loss of phylogenetic diversity. Finally, we identified biogeographic regions that are relatively species-poor but rich in evolutionary history, and suggest these regions deserve greater conservation priority. Our study provides phylogenetic information that is important for developing a unified management plan for mangrove ecosystems worldwide.

## Introduction

Preserving ecosystem services – the benefits humans derive directly and indirectly from nature (e.g. food production, plant pollination, medicinal plants, clean water, nutrient cycling, carbon sequestration, climate stability, recreation and tourism) – is a major challenge [1], [2]. Human welfare is intrinsically dependent on the sustainable delivery of such services; however, they are being rapidly eroded due to the unprecedented rate at which species – the service providers – are being lost through extinctions. For example, early studies suggested that rates of species loss might be about 1000–10 000 times greater than past extinction rates [3], [4] with particularly elevated rates in tropical biomes [5], a unique reservoir of life-form diversity.

To date, studies of extinction risk have tended to be focused on vertebrates (e.g. [6]–[14]). However, studies on terrestrial plants are becoming more commonplace (e.g. [15]–[18]). Although the taxonomic distribution of extinction risk is generally non-random [6], [7], [15], [16], the tree of animal life and that for plants are not pruned the same way. For instance, in vertebrates the majority of at-risk species are members of species-poor clades, and it has been suggested that their extinction would result in a disproportionate loss of evolutionary history [19], [20]. However, for terrestrial plants, extinction drivers appear to target particularly young and fast-evolving plant lineages [16], and at-risk species tend to fall within species-rich clades [21]; their extinction might therefore have a less pronounced impact on the plant phylogeny (but see [15]).

Whilst evidence suggests that many aquatic plants are highly threatened [22]–[26], and perform valuable ecosystem services [27], the phylogenetic ‘fingerprint’ of extinction risks in such systems, for example, the mangrove biome, has been less well studied. We know little about the forces relevant to community assembly in mangrove systems or about the phylogenetic basis of risk factors that predispose some mangrove species to higher extinction risk. A more detailed understanding of the phylogenetic structure of mangrove assemblages will aid in the development of management practices aimed at safeguarding their evolutionary future, and ensuring the sustainable delivery of ecosystem services [2], [28]. To date, predictive models of extinction risk at global scales tend to explain only a small amount of the variation in threat status (∼30% for mammals [10]; and ∼10% for tropical angiosperms [29]). It is therefore urgent that we work to improve our understanding of extinction risk, especially in understudied ecosystems, given current rates of species loss [1].

Mangroves have a tropical and subtropical distribution, and are linked to multiple ecosystem services (e.g. carbon sequestration and nutrient cycling [30]), act as keystone species [31], [32], and provide direct and indirect economic benefits (e.g. almost 80% of global fish catches are dependent to some extent on mangroves [33], [34], and indirect benefits may even be greater). Overall, the ecosystem services provided by mangrove forests are estimated to be worth at least US$1.6 billion per year worldwide [27], [35]. Given these large ecological and economic benefits, the recent findings of a global trend towards a reduction of range extent across mangrove biome due to human activities [24], [36] and climate change [37] is of major concern. It is estimated that we are losing 1–8% of mangrove cover each year [36], [38], [39], and that if current trends continue, the entire mangrove biome may be lost within the next 100 years [31]. One major consequence of this reduction in mangrove extent that we are already experiencing is the concomitant loss of associated species diversity – today, almost 40% of mangrove-dependent animal species are considered to be at higher risk of extinction [40].

In this study, we contrast the global distribution of mangrove species richness and threatened species richness with the distribution of phylogenetic diversity. We then construct alternative models of global decline (proportion of global population in decline) and extinction risk (derived from IUCN Red List categories) to identify key drivers of threat. We show that areas rich in evolutionarily unique species (subtending from long phylogenetic branches) match to those with highest global decline, and that human pressure and species ages are important predictors of risk of extinction in mangrove ecosystems.

## Results

**Figure 1:**
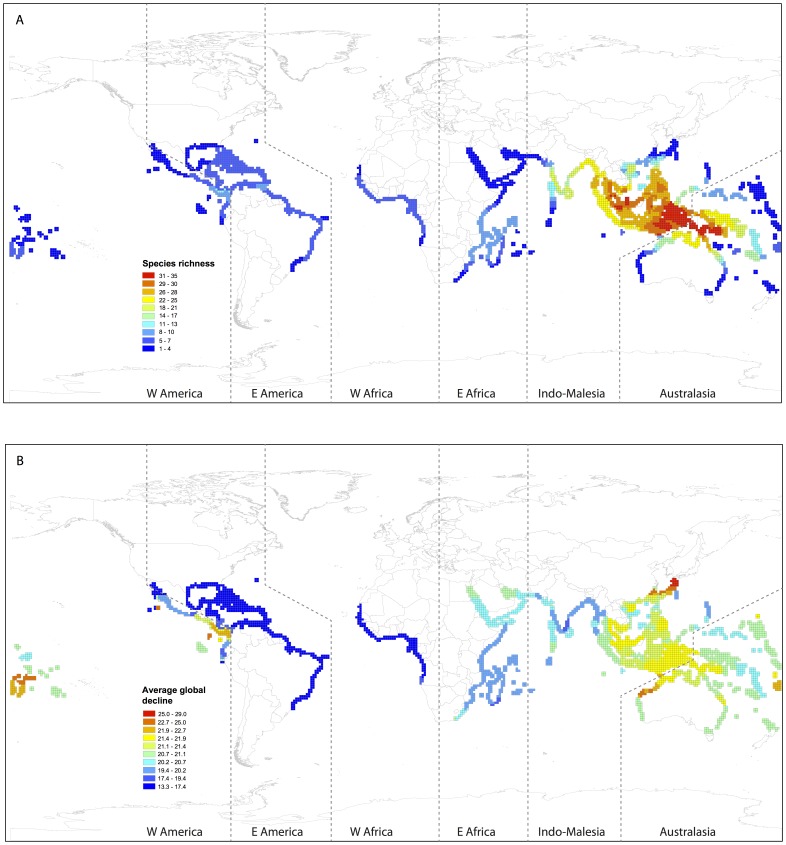
Geographical distribution of species richness (A) and global decline (see Materials and Methods) (B) in mangrove ecosystems across six biogeographical regions, per quarter degree squares (QDS).

**Figure 2:**
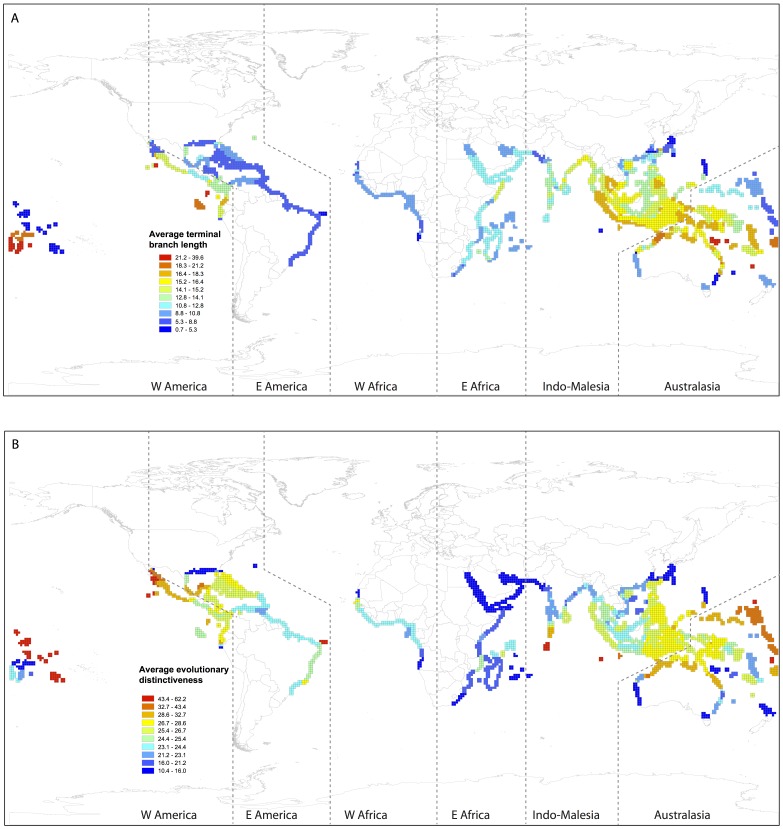
Geographical distribution of phylogenetic diversity within mangrove ecosystem for (A) mean terminal branch lengths, and (B) mean evolutionary distinctiveness across the same six biogeographical regions depicted inFigure 1.

Across the mangrove biome, we found that biogeographic regions of high species richness contained a high proportion of species in decline globally (mean decline in population size, see Materials and Methods) (Pearson correlation r = 0.38, p<0.001). The American West coast proved an exception to this general trend, with low species richness and high global decline (Figure 1A,B). Further, we compared the spatial distribution of mean global decline versus mean species terminal branch length (representing species evolutionary ages) and evolutionary distinctiveness (Figures 1B, 2A,B). Both species ages and evolutionary distinctiveness depict species phylogenetic uniqueness with the difference that the latter additionally accounts for evolutionary relationships deeper in the phylogenetic tree [41]. Species which are more evolutionarily distinct have few close relatives, whereas species descending from long terminal branch lengths are evolutionarily distant from their nearest phylogenetic neighbour, but might nonetheless fall within species rich clades. We found that only branch length was significantly correlated with global decline (Pearson correlation r = 0.46, p<0.0001) such that areas with older species (species subtending from longer branch lengths) tended to experience greater global decline (Figure 1B vs. Figure 2A).

We evaluated species evolutionary relatedness across the six mangrove biogeographic regions (West America, East America, West Africa, East Africa, Indo-Malesia and Australasia) using the net relatedness index (NRI) and the net taxon index (NTI). Using the NRI metric, we found a trend towards phylogenetic overdispersion such that species are, on average, less closely related to one another than expected by chance, but significantly so only along the East African and Indo-Malesian coasts (East America: NRI = −0.32, p = 0.59^ NS^; West Africa: NRI = −1, p = 0.79^ NS^; Australasia: NRI = −0.99, p = 0.82^ NS^; East Africa and Indo-Malesia:(NRI = −1.62 and −1.51, p<0.05* respectively). The American West coast again diverged from general trends, with a positive, but non-significant NRI (NRI = 0.04; p = 0.5^ NS^). Contrasting with the patterns observed for NRI, NTI, which better captures relationships towards the tips of the phylogeny [42], [43], indicated a tendency for closely related species to co-occur more often than expected by chance, and significantly so in four of the six biogeographic regions (West America: NTI = 2.87, p = 0.002**; East America: NTI = 2.59, p = 0.004**; West Africa: NTI = 1.62, p = 0.05^ NS^; East Africa: NTI = −0.68, p = 0.72^NS^; Indo-Malesia: NTI = 2.52, p = 0.005**; Australasia: NTI = 1.77, p = 0.039*).

**Figure 3:**
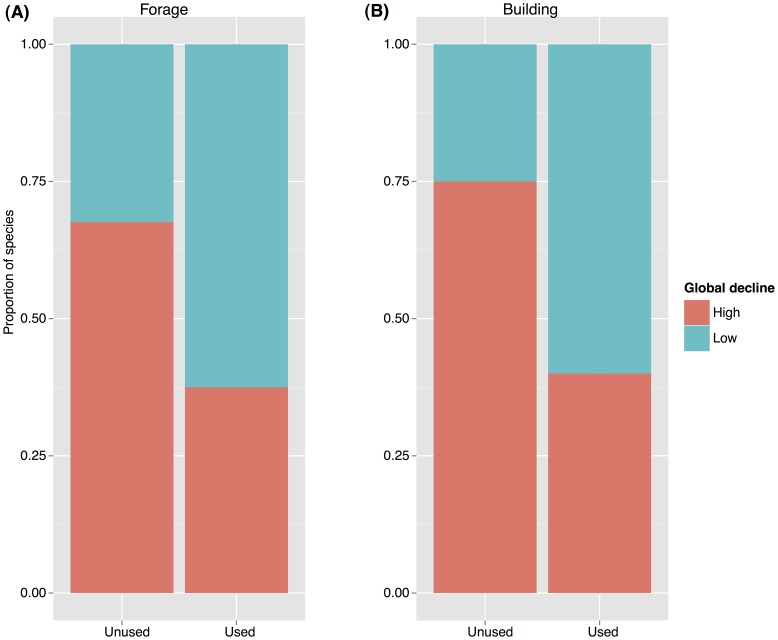
Stacked histograms of the proportion of species declining globally when used for (A) structural building, and (B) forage.

We found no evidence for phylogenetic signal in extinction risk as quantified by the IUCN Red List (K = 0.02, p = 0.93) or global decline (K = 0.05; p = 0.67; Figure S1). However, phylogenetically informed analyses are recommended even in the absence of phylogenetic signal in the response variable [44], we therefore used phylogenetic generalised least squares regressions (PGLS) to model global decline and extinction risk. In our univariate models, we found only species branch length (species age) was a significant predictor of global decline (p = 0.03*), such that older species tended to have greater global decline, explaining 10.16% of the total variation in declines (Table 1). In the multivariate model, species age becomes highly significant (p = 0.008**) but explanatory power increases only marginally (13.84%), and the covariates in the model remained non-significant (model p-value = 0.03*). In contrast, our models of extinction risk (IUCN threat category) identified human pressure as the single best predictor, but it was only significant when either species evolutionary distinctiveness or species age was also included into the model (p = 0.013*, r^2^ = 0.254, and p = 0.0087**, r^2^ = 0.145 for the multiple regression with species age and species evolutionary distinctiveness respectively, Table 2). However, we found that the relationship between extinction risk and human pressure was negative, such that species exposed to higher human pressure tended to have lower probability of extinction. Of the 16 uses examined, two, structural building and forage, showed an independent association with global decline although significance was marginal (building: χ^2^ = 5.3, df = 1, p = 0.02*; forage: χ^2^ = 3.0, df = 1, p = 0.08; Figure 3).

**Table 1:** Model coefficients for the PGLS models of global decline in mangrove ecosystems.

Univariate models (log_10_-transformed)	Predictors (log_10_-transformed)	P values	Multiple R-squared	Slope	Intercept
	Human pressure	0.098	0.0635	−0.3820	4.041
	BL	0.032	0.1016	0.0470	2.917
	ED	0.920	0.0002	−0.0063	3.036
	FD	0.920	0.0002	0.0285	2.948
	H_max_	0.530	0.0089	−0.0238	3.075
	Propagule size	0.820	0.0012	−0.0232	3.045
**Multivariate models (log_10_-transformed)**	**Predictors (log_10_-transformed)**	**P values**	**Adjusted R-squared**	**Slope**	**Intercept**
Global decline ∼ Propagule size+H_max_+BL+Human pressure (model p-value = 0.03)	Propagule size	0.920	0.1384	0.0103	4.0215
	H_max_	0.230		−0.0525	
	BL	0.008		0.0622	
	Human pressure	0.089		−0.3804	
Global decline ∼ Propagule size+H_max_+ED+Human pressure(model p-value = 0.5847)	Propagule size	0.951	−0.0224	0.00719	4.1876
	H_max_	0.770		−0.0127	
	ED	0.630		−0.0312	
	Human pressure	0.110		−0.3920	

BL, terminal branch length; ED, evolutionary distinctiveness; H_max_, maximum plant height. For all models the ML estimate of Lambda = 0.

**Table 2:** Model coefficients for the PGLS models of extinction risk in mangrove ecosystems.

(b) Extinction probabilityUnivariate models (log_10_-transformed)	Predictors (log_10_-transformed)	P values	Multiple R-squared	Slope	Intercept
	Human pressure	0.053	0.0841	−3.9970	1.467
	BL	0.250	0.0302	0.2320	−9.728
	ED	0.390	0.0168	−0.4870	−7.698
	FD	0.230	0.0321	−3.0890	−1.929
	H_max_	0.088	0.0661	0.5890	−10.708
	Propagule size	0.320	0.0228	0.9200	−10.395
**Multivariate models (log_10_-transformed)**	**Predictors (log_10_-transformed)**	**P values**	**Adjusted R-squared**	**Slope**	**Intercept**
extinction probability ∼ Propagule size+H_max_+BL+Human pressure (model p-value = 0.04)	Propagule size	0.740	0.1254	0.3203	2.2520
	H_max_	0.110		0.6483	
	BL	0.540		0.1238	
	Human pressure	0.013		−5.1415	
extinction probability ∼ Propagule size+H_max_+ED+Human pressure (model p-value = 0.03 )	Propagule size	0.890	0.145	0.1361	5.5950
	H_max_	0.057		0.7253	
	ED	0.260		−0.6234	
	Human pressure	0.009		−5.5424	

BL, terminal branch length; ED, evolutionary distinctiveness; H_max_, maximum plant height. For all models the ML estimate of Lambda = 0.

## Discussion

We are currently witnessing a mass extinction event on a scale similar to that of the paleontological past [4], [45], [46]. Here, we explored patterns of range contraction and extinction risk in mangroves, an aquatic forest biome widely distributed across the tropics. We revealed that regions with a high proportion of species experiencing population declines, specifically, Indo-Malesia and Australasia, which represent centres of mangrove species diversity, also correspond to areas particularly rich in evolutionarily distinct species (old species subtending from long phylogenetic branch lengths). The central West coast of America is unusual in that it is relatively poor in species diversity but rich in species subject to high global decline.

The global geographical distribution of mangroves is dictated by several environmental and historical factors [47]. Early studies suggested a range restriction of mangroves to regions where mean air temperatures of the coolest months are higher than 20°C and the seasonal temperature fluctuation does not exceed 10°C [48]–[50]. Additionally, limitations to propagule dispersal, for example, due to barriers imposed by wide expanses of water, and major continental landmasses likely further restrict movement of species between biogeographical regions. Given such limitations, we might expect species within geographical regions to be largely a product of *in situ* diversification, representing clusters of closely related species on the phylogenetic tree of mangroves.

We evaluated the evolutionary relatedness among mangrove species within biogeographical regions. However, we did not detect significant phylogenetic clustering, but rather we found that most mangrove assemblages do not differ from random expectations, whilst mangrove species along the East African and Indo-Malesian coastlines were less closely related to each other than expected by chance. Our results indicate that regional mangrove species assemblages are not simply explained by diversification in the presence of strong geographical barriers and/or environmental filtering. Random patterns may arise from competing processes offsetting one another, for example, environmental attraction versus competitive repulsion (see [51]), and/or frequent dispersal between biogeographic regions. Our observation for phylogenetic over-dispersion in Eastern Africa might also be a product of complex interacting forces, including facilitation [52], competition [42], [53] and biotic interchange [54], but more data are required to fully evaluate assembly mechanisms. Nonetheless, evidence of significant clustering towards the tips of the phylogeny (as indicated by NTI), perhaps captures the signature of more recent *in situ* diversification.

Because we find evidence that the most closely related species tend to be found within the same biogeographical realms, we might expect that they would also share similar risk of extinction, which should translate to phylogenetic signal in extinction risk and global decline. However, we found no evidence for phylogenetic signal in either threat metric (IUCN Red List status or global decline), rather, our results indicate that threat is randomly distributed across the phylogeny. The loss of evolutionary history might be relatively low under random extinction [55], although contingent upon the underlying tree topology [56]. However, we observed that areas with a high proportion of species experiencing global declines correspond to areas of unique evolutionary history, suggesting that whilst extinction risk might not demonstrate strong phylogenetic structure, the loss of currently threatened species might still have a disproportionate impact on mangrove phylogenetic diversity regionally. The loss of phylogenetic diversity may be of concern because it captures the functional and ecological diversity represented along the branches of the tree-of-life, and has been linked to ecosystem function (e.g. [57], [58]) and stability [57]. As the tree-of-life is pruned through extinctions we would then lose these services associated with the functional and ecological diversity represented along its branches.

We evaluated various predictors of threat, including plant height, propagule size, human pressure, and two evolutionary variables, species age and evolutionary distinctiveness. We found that species age was a significant correlate of global decline whilst human pressure was the best predictor of extinction risk. In all cases, less than 15% of the variation in species threat was explained, suggesting other factors not included in our model must be important in determining species declines. Our findings were somewhat surprising in that we did not find any evidence linking morphological characters (i.e. plant height and propagule size) to threat status; other life-history traits might therefore be missing in our models.

Contrary to expectations, we found species exposed to higher human pressure (here defined as the total number of uses per species) had lower probability of extinction. The negative correlation between human activities and extinction risk may be linked to our definition of human pressure. We considered the total number of uses recorded for each species as indicative of the level of human pressure. Such surrogacy may be misleading: for instance, a species known to fill one common need in a given area may be subject to greater pressure than species with multiple but less extractive uses. Further, it is likely that our list of uses is not comprehensive, and some important uses might not be included despite our best efforts. In addition, we separately tested the influence of each use on global decline. We found that only uses associated with building and forage show strong relationships on their own. It is possible that species which are currently more common (i.e. those that have not yet declined) are preferably utilised for more intensive building and forage purposes (Figure 3), than species that are already in decline. Whilst such species are not threatened now, if current trends continue, the negative correlations might turn positive in the future.

In addition, we found that older species tended to be more threatened, in contrast to recent finding for terrestrial plants [16]. There are several explanations for why extinction risk is greater for older species. First, the ‘taxon cycle’ of Wilson [59] predicts that older species should have higher extinction probabilities as species expand and contract in their geographical distributions over their evolutionary lifetimes. Second, the trend for higher risk in older species might reflect the pattern of historical extinctions, in which older species represent survivors of once more diverse clades [7]. Third, it is possible that older species are for some reason less well suited to the ongoing changes in environment, resulting in greater risk.

Mangrove species have evolved a unique suite of specialisations to tidal environments [60], where they provide important ecosystem services [24]. A comprehensive species-level analysis of richness and extinction risk has recently been conducted [24]. Our study adds to this body of work information on the phylogeography of mangrove forests and the likely phylogenetic consequences of potential extinctions in these ecosystems. Specifically, our study indicates that species evolutionary history may be an important predictor of extinction risk, although the underlying mechanisms remain to be identified. We observe a worrying overlap between regions in which species are undergoing declines and regions rich in evolutionarily distinct species. We suggest that to safeguard the provisioning of the many and valuable ecosystem services provided by mangrove forests, conservation efforts should focus not only on preserving species, but also upon maintaining their evolutionary diversity.

## Materials and Methods

### Sampling, Morphological Traits and Human Pressure Data

Mangrove forests comprise 70 species widely distributed in tropical and subtropical regions. Recent studies have explored the patterns of IUCN categories and global decline among mangrove species [24], [47] distributed across six biogeographical coastal regions: West America, East America, West Africa, East Africa, Indo-Malesia and Australasia ([61]; Figures 1 and 2). Here we focus on the phylogenetic ecology of mangrove ecosystems, and included in our study the 54 species for which DNA sequences are available, thereby allowing us to construct a robust phylogenetic tree depicting evolutionary relationships within the group.

We recorded from the literature two plant life-history traits, plant maximum height and propagule size, which are linked to plant dispersal ability [24], [47], [60], [62]. Our measures of extinction risk and global decline follow Polidoro et al. [24], and are derived from IUCN Red List categories, and comprehensive data on mangrove taxonomy, distribution, population trends, ecology, life-history traits, past and current threats, and conservation actions for each species. In addition, we recorded the different uses associated with each mangrove species, including firewood and charcoal, building and structural, carving, cultural, spiritual, food, forage and fodder, medicinal, ornamental, shade or chemical compounds, and medicinal [34], [35], [60], [63]–[74]. We used the total number of uses recorded for each species as an indirect measure of human pressure on that species.

### Phylogeny Reconstruction and Divergence Time Estimation

We retrieved from GenBank/EBI DNA sequences for three genes, *rbcL*, ITS and 18S (see Table S1 for accession numbers) for 54 of the 70 mangrove species. DNA sequences were aligned using Multiple Sequence Comparison by Log-Expectation (MUSCLE v.3.8.31; [75]) and manually edited. The aligned sequences were then concatenated in a single matrix. Phylogeny reconstruction was performed using BEAST v.1.7.4 assuming a relaxed-clock model [76] and a GTR+I+Γ model of sequence evolution for each partition, selected based on Akaike information criterion (AIC) using Modeltest v.2.3 [77]. The tree prior was estimated assuming a speciation model following a Yule process with an uncorrelated lognormal model for rate variation among branches. For calibration purpose, we added to the mangrove matrix members of the families Oleaceae, Moraceae, Malvaceae and Vitaceae. These families were used as secondary calibration points with a normal prior distribution based on Bell et al. [78] as follows: Oleaceae crown node (41 Ma, SD 6 Ma), Moraceae crown node (31 Ma, SD 4 Ma), Malvaceae crown node (39 Ma, SD 4 Ma), Vitaceae crown node (43 Ma, SD 9 Ma), angiosperm crown (149 Ma, SD 3 Ma). We included *Amborella trichopoda* and *Nymphaea alba* as outgroups, following Schneider et al. [79]. Monte Carlo Marcov Chains were run for 100 million generations sampling every 1000 generations. Convergence was checked using Tracer v.1.5, and of the resulting 100001 trees, we removed 15000 as burnin, the remaining 85001 trees were combined using treeAnnotator v.1.7.1.

### Data Analysis

First, for each of the mangrove species represented in the phylogenetic tree, we extracted range map data from the IUCN Red List database (http://www.iucnredlist.org). Species distributions were then projected onto a Behrmann equal-area cylindrical projection in ArcMap v.10, and gridded at a resolution of 0.25°×0.25° (approximately 27.5×27.5 km at the equator). We then generated a series of maps to capture spatial variation in species richness (SR), global decline, species evolutionary distinctiveness (ED), and mean species age. Species richness simply captures the number of species occupying a grid cell. Global decline represents information on the mean decline in population size of each species and was recorded from Polidoro et al. [24]. Evolutionary distinctiveness was calculated using the R library Picante [80]. This metric partitions branch lengths by the total number of species subtending it and was evaluated using the function ‘evol.distinct’ based on fair proportions [41]. Species ages were calculated as the terminal branch length (BL) connecting each species to the phylogeny, and captures species evolutionary uniqueness (unshared branch lengths).

Second, we evaluated the phylogenetic structure within biogeographic regions using the net relatedness index (NRI) and net taxon index (NTI; [42], [43]) to compare observed pair-wise phylogenetic distances against expectations from random species assemblage. This comparison provides insights into community assembly mechanisms (e.g. competition vs. habitat filtering, [42]) within mangrove ecosystems. For this purpose, we used all mangrove species included in our phylogenetic tree as the regional pool (null model "phylogeny.pool" in R library Picante [80]). NRI describes a tree-wide pattern of dispersion, whereas NTI is more sensitive to phylogenetic structure towards the tips of the phylogeny. A strong negative value of NRI or NTI indicates that communities are composed of less related species whereas a strong positive value indicates clustering of closely related species. Values of NRI and NTI that do not depart significantly from zero indicate random species assemblages [42].

Finally, we tested for a phylogenetic signal in extinction risk and global decline using Blomberg’s K statistics [81], and constructed a series of regression models to explore predictors of threat using phylogenetic GLS models [82], [83]. Extinction risk and global decline data were recorded from Polidoro et al. [24] and converted into extinction probabilities using the extinction probability IUCN50 (see [84]). Regression models were generated using the function ‘pgls’ implemented in the R package ‘Caper’ [85]. For both extinction risk and global decline, we evaluated four explanatory variables: plant maximum height, propagule size (measured as propagule volume recorded from Duke et al. [46]), human pressure (measured as total number of uses recorded for each species), and species ages or species evolutionary distinctiveness (see above). In addition, we explored separately whether different uses had varying levels of impact on global decline using Pearson's Chi-squared test with Yates' continuity correction.

## Supporting Information

Figure S1 **The 50% majority rule consensus tree showing distribution of global decline within mangrove species obtained from a Bayesian analysis of the combined dataset (** ***rbcL*** **+ITS +18S).** Numbers above branches are posterior probability above 50%. Outgroups and taxa used for calibration were pruned from the tree prior to further analyses.
(TIFF)

Table S1 **Voucher table with GenBank accession numbers for the gene regions used in phylogeny reconstruction, IUCN categories, extinction probabilities, global decline, life history traits (maximum plant height and propagule size), and human pressure for the mangrove species included in our analysis.**
(DOC)
