# Supplementary figures and images for: A Global Trend towards the Loss of Evolutionarily Unique Species in Mangrove Ecosystems
Source: PLoS One. 2013 Jun 21;8(6):e66686. doi: 10.1371/journal.pone.0066686 (PMC3689665; doi:10.1371/journal.pone.0066686)

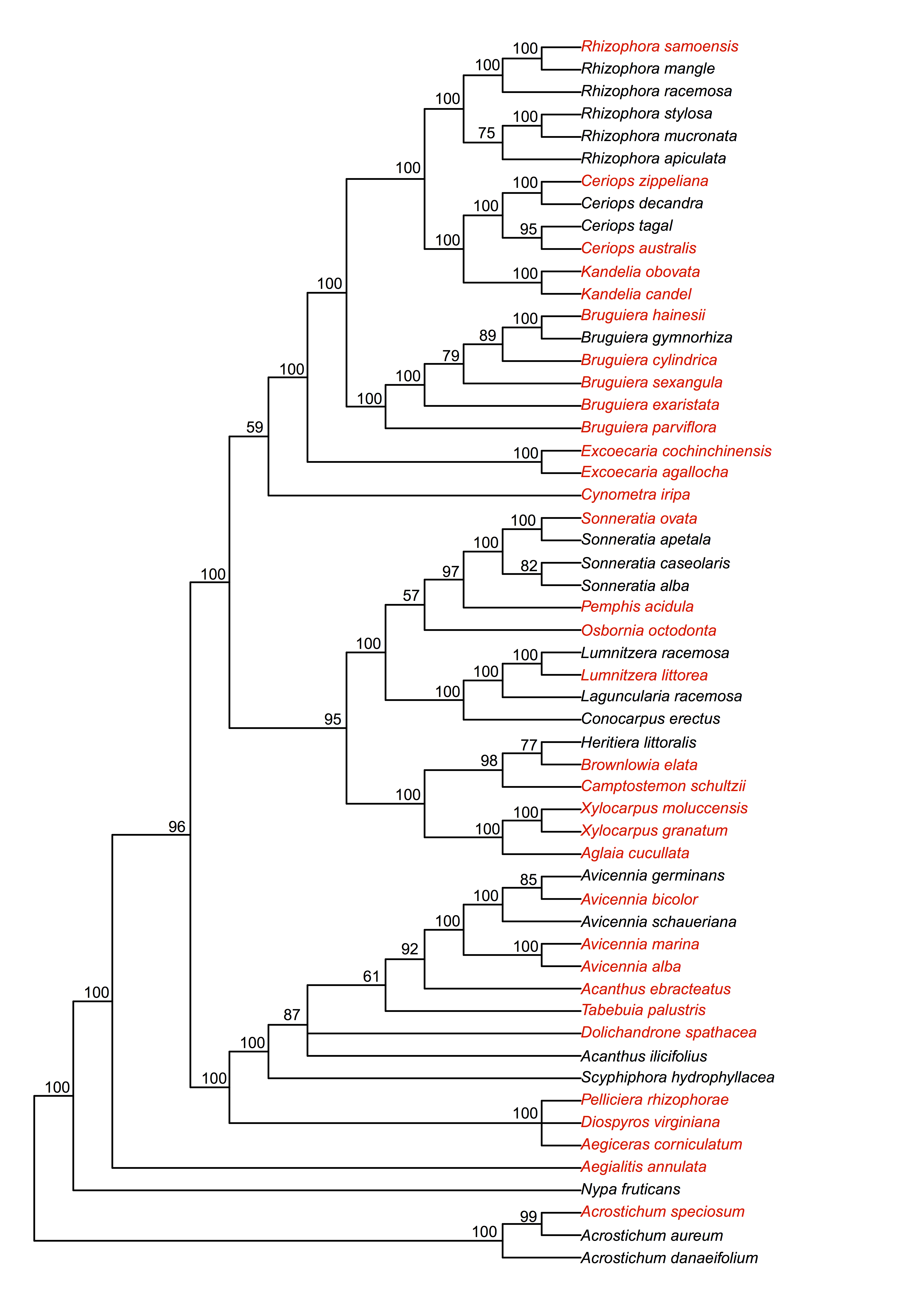

Supplement: Figure S1 — The 50% majority rule consensus tree showing distribution of global decline within mangrove species obtained from a Bayesian analysis of the combined dataset ( rbcL +ITS +18S). Numbers above branches are posterior probability above 50%. Outgroups and taxa used for calibration were pruned from the tree prior to further analyses. (TIFF) [file pone.0066686.s001.tiff]
