# Supplementary material for: A Global Trend towards the Loss of Evolutionarily Unique Species in Mangrove Ecosystems
Source: PLoS One. 2013 Jun 21;8(6):e66686. doi: 10.1371/journal.pone.0066686 (PMC3689665; doi:10.1371/journal.pone.0066686)
Supplement: Table S1 — Voucher table with GenBank accession numbers for the gene regions used in phylogeny reconstruction, IUCN categories, extinction probabilities, global decline, life history traits (maximum plant height and propagule size), and human pressure for the mangrove species included in our analysis. (DOC) [file pone.0066686.s002.doc]

| **APG III (2009) Family** | **Scientific name** | **IUCN status** | **Red List criteria version** | **Year assessed** | **GenBank accessions** | | | **ED** | **BL** | **Extinction probability** | **Human pressure** | **H_max_** | **Propagule size** | **Global decline** |
| --- | --- | --- | --- | --- | --- | --- | --- | --- | --- | --- | --- | --- | --- | --- |
|  |  |  |  |  | **18S** | ***rbcL*** | **ITS** |  |  |  |  |  |  |  |
| Acanthaceae | *Acanthus ebracteatus* Vahl | LC |  |  | AY289642 | AY289682 |  | 18.185 | 0.549 | 0.00005 | 7 | 1.5 | 3 | 22 |
|  | *Acanthus ilicifolius* L. | LC | 3.1 | 2011 |  |  | GQ141552 | 25.240 | 0.549 | 0.00005 | 1 | 1.5 | 3 | 20 |
| Amborellaceae | *Amborella trichopoda* Baill. |  |  |  | U42497 | L12628 |  |  |  |  |  |  |  |  |
| Arecaceae | *Nypa fruticans* Wurmb | LC | 3.1 | 2010 | AY289649 | M81813 |  | 36.203 | 12.087 | 0.00005 | 4 | 9 | 5 | 20 |
| Avicenniaceae | *Avicennia alba* Blume | LC |  |  |  | AY008831 | EF540977 | 12.0614 | 2.125 | 0.00005 | 5 | 20 | 3 | 24 |
|  | *Avicennia bicolor* Standl. | VU |  |  |  | AY008829 | EF540989 | 10.397 | 2.125 | 0.05 | 1 | NA | NA | 31 |
|  | *Avicennia germinans* (L.) L. | LC | 3.1 | 2010 |  | AY008830 | DQ469860 | 10.397 | 7.410 | 0.00005 | 8 | 25 | 4 | 17 |
|  | *Avicennia marina* (Forssk.) Vierh. | LC | 3.1 | 2010 | AY289641 | AY289681 | EF540978 | 12.061 | 10.157 | 0.00005 | 9 | 14 | 4 | 21 |
|  | *Avicennia schaueriana* Stapf & Leechm. ex Moldenke | LC |  |  |  |  | EF540986 | 11.202 | 1.0243 | 0.00005 | 0 | NA | NA | 6 |
| Bignoniaceae | *Dolichandrone spathacea* (L.f.) Seem. | LC |  |  | AY289643 | AY289683 |  | 25.240 | 19.276 | 0.00005 | 9 | 25 | 5 | 23 |
|  | *Tabebuia palustris* Hemsl. | VU |  |  |  | JQ590857 |  | 25.797 | 2.809 | 0.05 | 0 | NA | NA | 33 |
| Combretaceae | *Conocarpus erectus* L. | LC |  |  | AY289636 | AF281477 | AY050562 | 49.908 | 14.392 | 0.00005 | 5 | 20 | 3 |  |
|  | *Laguncularia racemosa* (L.) C.F.Gaertn. | LC | 3.1 | 2010 | AY289635 | FJ381826 | AF425685 | 46.432 | 0.7122 | 0.00005 | 6 | 18 | 3 | 17 |
|  | *Lumnitzera littorea* (Jack) Voigt | LC |  |  | AY289637 | AF425718 | AF160468 | 36.367 | 11.942 | 0.00005 | 5 | 15 | 3 | 22 |
|  | *Lumnitzera racemosa* Willd. | LC |  |  |  | FJ381827 | AF160467 | 36.367 | 21.075 | 0.00005 | 4 | 10 | 3 | 19 |
| Ebenaceae | *Diospyros littorea* (R.Br.) Kosterm. | LC |  |  |  | EU980774 | DQ499076 |  |  |  |  |  |  |  |
| Euphorbiaceae | *Excoecaria agallocha* L. | LC |  |  | AY289628 | AY794839 |  | 25.956 | 39.561 | 0.00005 | 5 | 20 | 3 | 21 |
|  | *Excoecaria indica* (Willd.) Müll.Arg. | DD |  |  | AB233573 | AB233885 | GU441816 |  |  |  |  |  |  |  |
| Fabaceae | *Cynometra iripa* Kostel. | LC |  |  | AY289630 | AY289677 |  | 47.651 | 32.043 | 0.00005 | 4 | 15 | 3 | 21 |
| Lamiaceae | *Clerodendrum inerme* (L.) Gaertn. |  |  |  | AY289644 | AY289684 | U77752 |  |  |  |  |  |  |  |
| Lythraceae | *Pemphis acidula* J.R. Forst. | LC |  |  | AY289639 | AY036138 | AY035762 | 62.185 | 2.301 | 0.00005 | 4 | 6 | 3 | 21 |
|  | *Sonneratia alba* Sm. | LC |  |  |  | GQ122038 | AY680875 | 31.648 | 4.0311 | 0.00005 | 5 | 15 | 4 | 20 |
|  | *Sonneratia apetala* Buch.-Ham. | LC |  |  |  |  | AF163697 | 33.453 | 0.421 | 0.00005 | 5 | 20 | 4 | 7 |
|  | *Sonneratia caseolaris* (L.) Engl. | LC |  |  |  | AY036143 | AF420219 | 31.648 | 0.421 | 0.00005 | 6 | 10 | 5 | 20 |
|  | *Sonneratia ovata* Backer | NT |  |  | AY289638 | AY036143 | AF163702 | 33.453 | 67.133 | 0.004 | 5 | 20 | 5 | 28 |
|  | *Abroma augustum* (L.) L. f |  |  |  |  | AJ012208 | AJ277462 |  |  |  |  |  |  |  |
|  | *Brownlowia tersa* (L.) Kosterm. | NT |  |  |  | AJ233147 |  |  |  |  |  |  |  |  |
|  | *Camptostemon schultzii* Mast. | LC |  |  |  | AF022120 | HQ658360 | 43.584 | 31.289 | 0.00005 | 5 | 30 | 2 | 24 |
|  | *Heritiera littoralis* Aiton | LC |  |  | AY289633 | AY082358 | AY083659 | 29.762 | 21.616 | 0.00005 | 2 | 30 | 5 | 20 |
| Meliaceae | *Aglaia cucullata* (Roxb.) Pellegr. | DD |  |  |  | JF738768 | AY695572 |  |  |  |  |  |  |  |
|  | *Xylocarpus granatum* J. Koenig | LC |  |  | AY289634 | AY289680 |  | 24.337 | 22.939 | 0.00005 | 7 | 22 | 6 | 21 |
|  | *Xylocarpus moluccensis* (Lam.) M. Roem. | LC |  |  |  | DQ238071 | FJ518907 | 24.337 | 28.152 | 0.00005 | 4 | 22 | 5 | 21 |
| Moraceae | *Bagassa guianensis* Aubl. |  |  |  |  | JQ625997 | FJ917001 |  |  |  |  |  |  |  |
|  | *Brosimum alicastrum* Sw. |  |  |  |  | AF500346 |  |  |  |  |  |  |  |  |
|  | *Castilla elastica* Cerv. |  |  |  |  | AF500348 | FJ916997 |  |  |  |  |  |  |  |
|  | *Naucleopsis guianensis* (Mildbr.) C.C. Berg |  |  |  |  | JQ626013 | FJ037848 |  |  |  |  |  |  |  |
| Myrsinaceae | *Aegiceras corniculatum* (L.) Blanco | LC |  |  | AY671951 | U96653 | FJ976669 | 33.095 | 0.409 | 0.00005 | 2 | 7 | 3 | 21 |
| Myrtaceae | *Osbornia octodonta* F.Muell. | LC |  |  |  |  | EF041844 | 77.010 | 12.087 | 0.00005 | 3 | 5 | 2 | 23 |
| Nymphaeaceae | *Nymphaea alba* L. |  |  |  |  | HM850203 | GU222363 |  |  |  |  |  |  |  |
| Oleaceae | *Fontanesia phillyreoides* Labill. |  |  |  |  | DQ673293 | AF534816 |  |  |  |  |  |  |  |
|  | *Jasminum simplicifolium* subsp. *suavissimum* (Lindl.) P.S.Green |  |  |  | AJ236035 | L01929 |  |  |  |  |  |  |  |  |
|  | *Ligustrum vulgare* L. |  |  |  |  | HQ384901 | EU314901 |  |  |  |  |  |  |  |
|  | *Osmanthus americanus* (L.) A.Gray |  |  |  |  | DQ673311 | EF362761 |  |  |  |  |  |  |  |
|  | *Picconia azorica* (Tutin) Knobl. |  |  |  |  | HM850258 |  |  |  |  |  |  |  |  |
| Plumbaginaceae | *Aegialitis annulata* R.Br. | LC |  |  | AY289640 | AJ312252 |  | 40.697 | 0.409 | 0.00005 | 1 | 3 | 3 | 24 |
| Pteridaceae | *Acrostichum aureum* L. | LC |  |  |  | AB574794 |  | 15.423 | 4.584 | 0.00005 | 6 | 2 | 1 | 19 |
|  | *Acrostichum danaeifolium* (Fée) C. Presl | LC |  |  |  | EF452129 |  | 18.805 | 4.993 | 0.00005 | 1 | 4 | 3 | 17 |
|  | *Acrostichum speciosum* (Fée) C. Presl | LC |  |  |  | AB246707 |  | 15.423 | 23.272 | 0.00005 | 6 | 2 | 3 | 21 |
| Rhizophoraceae | *Bruguiera cylindrica* (L.) Blume | LC |  |  |  | AF127694 | HM366080 | 13.0766 | 26.946 | 0.00005 | 4 | 10 | 3 | 24 |
|  | *Bruguiera exaristata* Ding Hou | LC |  |  |  | AF127695 | AF130335 | 13.349 | 8.103 | 0.00005 | 0 | NA | NA | 23 |
|  | *Bruguiera gymnorhiza* (L.) Lam. | LC | 3.1 | 2010 | AF206875 | DQ659092 | HM366108 | 11.0590 | 4.369 | 0.00005 | 8 | 30 | 4 | 20 |
|  | *Bruguiera hainesii* C.G.Rogers | CR |  |  |  |  | EU000399 | 11.0590 | 4.369 | 0.97 | 1 | 33 | 3 | 27 |
|  | *Bruguiera parviflora* (Roxb.) Wight & Arn. ex Griff. | LC |  |  |  | AF127692 | HM366110 | 27.587 | 44.917 | 0.00005 | 4 | 25 | 3 | 21 |
|  | *Bruguiera sexangula* (Lour.) Poir. | LC |  |  | AY289626 | AF127691 | HM366136 | 19.138 | 31.289 | 0.00005 | 5 | 20 | 4 | 21 |
|  | *Ceriops australis* (C.T.White) Ballment, T.J.Sm. & J.A.Stoddart | LC |  |  |  | AF127683 | EF118998 | 12.910 | 37.435 | 0.00005 | 3 | 10 | 3 | 24 |
|  | *Ceriops decandra* (Griff.) W.Theob. | NT |  |  |  |  | EF119014 | 14.804 | 16.711 | 0.004 | 6 | 35 | 4 | 12 |
|  | *Ceriops tagal* (Perr.) C.B.Rob. | LC |  |  | AY289624 | AF127684 | AF105083 | 12.910 | 2.537 | 0.00005 | 5 | 40 | 4 | 18 |
|  | *Ceriops zippeliana* Blume | LC |  |  |  |  | EF119070 | 14.804 | 2.537 | 0.00005 | 4 | NA | NA | 23 |
|  | *Kandelia candel* (L.) Druce | LC |  |  | AY289625 | AF127682 | AF105081 | 13.228 | 2.264 | 0.00005 | 3 | 7 | 4 | 23 |
|  | *Kandelia obovata* Sheue, H.Y. Liu & J. Yong | LC |  |  |  |  | EU000402 | 13.228 | 2.323 | 0.00005 | 1 | NA | NA | 29 |
|  | *Rhizophora apiculata* Blume | LC |  |  |  | AF127685 | HQ337923 | 17.613 | 21.110 | 0.00005 | 6 | 20 | 5 | 20 |
|  | *Rhizophora mangle* L. | LC | 3.1 | 2010 |  | JX664070 | HQ337957 | 13.139 | 19.197 | 0.00005 | 7 | 20 | 5 | 17 |
|  | *Rhizophora mucronata* Lam. | LC | 3.1 | 2010 |  | AF127687 | HQ337953 | 13.047 | 13.849 | 0.00005 | 8 | 25 | 5 | 20 |
|  | *Rhizophora racemosa* G.Mey. | LC |  |  |  | AF127690 | HQ337960 | 15.782 | 13.849 | 0.00005 | 1 | NA | NA | 15 |
|  | *Rhizophora samoensis* (Hochr.) Salvoza | NT |  |  |  |  | HQ337959 | 13.139 | 35.919 | 0.004 | 2 | 20 | 5 | 29 |
|  | *Rhizophora stylosa* Griff. | LC |  |  | AY289627 | AF127686 | HQ337941 | 13.047 | 48.600 | 0.00005 | 4 | 5 | 5 | 20 |
| Rubiaceae | *Scyphiphora hydrophylacea* C.F.Gaertn. | LC |  |  | AY289646 | EU817432 |  | 44.413 | 4.0311 | 0.00005 | 7 | 3 | 2 | 20 |
| Tetrameristaceae | *Pelliciera rhizophorae* Planch. & Triana | VU |  |  | AF206983 | AF421099 | AY452670 | 43.237 | 42.101 | 0.05 | 3 | 15 | 5 | 27 |
| Vitaceae | *Ampelocissus thyrsiflora* (Blume) Planch. |  |  |  |  | AJ402919 |  |  |  |  |  |  |  |  |
|  | *Ampelopsis megalophylla* Diels & Gilg |  |  |  |  | AJ402920 |  |  |  |  |  |  |  |  |
|  | *Vitis rotundifolia* Michx. |  |  |  |  | AF119174 |  |  |  |  |  |  |  |  |
